# Evaluation of gestational age by pregnancy outcomes and distribution of pregnancy-related codes in Korean claims data

**DOI:** 10.4178/epih.e2026007

**Published:** 2026-02-04

**Authors:** Woo-Jung Kim, Yunha Noh, Yongtai Cho, Eun-Young Choi, HyunJoo Lim, Hyesung Lee, Ju-Young Shin

**Affiliations:** 1 Department of Pharmaceutical Industry, Sungkyunkwan University School of Pharmacy, Suwon, Korea; 2 College of Pharmacy, Chonnam National University, Gwangju, Korea; 3 Department of Pharmacy, Sungkyunkwan University School of Pharmacy, Suwon, Korea; 4 Department of Biohealth Regulatory Science, Sungkyunkwan University, Suwon, Korea; 5 Department of Medical Informatics, Kangwon National University College of Medicine, Chuncheon, Korea; 6 Department of Clinical Research Design & Evaluation, Samsung Advanced Institute for Health Sciences and Technology, Sungkyunkwan University, Seoul, Korea

**Keywords:** Gestational age, Pregnancy outcomes, Algorithms, Claims data, Prenatal tests, Korea

## Abstract

**OBJECTIVES:**

This study aimed to evaluate a fixed-duration algorithm for gestational age (GA) estimation according to pregnancy outcomes and to describe the GA distribution of pregnancy-related codes in Korea.

**METHODS:**

We included 351,055 pregnancy episodes (2019–2022) from linked data between the National Health Insurance Service and the Korea Immunization Registry Information System (KIRIS). GA from claims data was estimated by subtracting fixed durations from the delivery date (algorithm-based GA), and GA derived from KIRIS was defined as the gold standard. Accuracy was evaluated as the proportion of episodes in which the difference between the estimated GA and the reference standard fell within ±2 weeks. We described the distributions of the GA at which each prenatal test, pregnancy complication, and diagnostic code was recorded.

**RESULTS:**

Algorithm-based GA estimation showed high accuracy for live births (92.2% within ±2 weeks) but markedly lower accuracy for non-live birth outcomes, including stillbirth (3.3%), termination (7.2%), spontaneous abortion (45.2%), and ectopic pregnancy (20.0%). In additional analyses aimed at identifying potential indicators for improving GA estimation, most events occurred within clinically expected timeframes, although some individual codes exhibited poor temporal alignment.

**CONCLUSIONS:**

Algorithm-based GA estimation using claims data performed well for live births but demonstrated limited accuracy for non-live birth outcomes. Incorporating information from prenatal tests and pregnancy complications may enhance GA estimation.

## GRAPHICAL ABSTRACT

[Fig f3-epih-48-e2026007]

## Key Message

Using nationwide linked claims and immunization registry data in Korea, this study systematically validated the performance of a fixed-duration algorithm for gestational age estimation across pregnancy outcomes. While high accuracy was observed for live births (92.2% within ±2 weeks), substantially poorer performance was identified for non-live birth outcomes, indicating marked outcome-specific heterogeneity. Integration of time-sensitive clinical indicators, including prenatal tests and pregnancy complications, may enhance the validity of gestational age estimation in administrative data research.

## INTRODUCTION

Gestational age (GA) estimation is essential for maternal and perinatal research, as it provides critical context for analyzing fetal development, pregnancy outcomes, and drug safety during pregnancy. In many epidemiologic studies using administrative data, clinically recorded GA is often unavailable [1]. To address this limitation, researchers frequently apply deterministic algorithms that estimate the last menstrual period by subtracting fixed gestational durations from pregnancy outcome dates, with 270–280 days being a typical assumption for term deliveries [1]. Non-live birth outcomes encompass various pregnancy types and occur across a wide gestational range. These algorithms are widely adopted in pharmacoepidemiologic studies [2,3], but validation has been limited, particularly outside Western countries [4,5].

Several studies in North America and Europe have demonstrated that fixed-duration algorithms can accurately estimate GA at term births, with more than 95% of algorithm-based estimates falling within two weeks of clinical records when using 39 weeks or 35 weeks for term and preterm births, respectively [1,3]. Previous studies have reported statistically significant but small differences in gestational length across racial and ethnic groups [6,7]. Although modest, such variation may meaningfully affect GA estimation and thereby introduce potential bias in perinatal research. Moreover, these findings may not be generalizable to other countries due to differences in coding practices and healthcare systems [8]. Furthermore, fixed-duration algorithms are less reliable for pregnancies ending in spontaneous or induced abortion or stillbirth, where clinical indicators are sparse and gestational durations are variable [9]. Previous validation studies conducted in North America and Europe have shown that fixed-duration algorithms generally achieve high accuracy for live births, with approximately 90–95% of GA estimates falling within ±1–2 weeks of clinical records [1-3,5]. In contrast, accuracy for non-live birth outcomes—such as spontaneous abortion, induced abortion, and stillbirth—tends to be substantially lower, often below 30–50%, as demonstrated in recent validation studies using large administrative or linked datasets [9-11]. These international findings highlight the need to evaluate whether similar performance patterns apply in Korean claims data, where coding practices and healthcare delivery systems differ from those of Western countries.

This study aims to provide empirical evidence for validating GA estimation methods in Korean administrative data and to offer insights into potential enhancements through clinical code profiling by evaluating a fixed-duration algorithm for GA estimation according to pregnancy outcomes and by describing the GA distribution of pregnancy-related codes in Korea.

## MATERIALS AND METHODS

### Data source

This study utilized two distinct databases: (1) the Korea Immunization Registry Information System (KIRIS) (from October 15, 2019 to March 31, 2022) and (2) the National Health Insurance Service (NHIS) claims database (from January 1, 2018 to June 30, 2022). The KIRIS contains records of maternal influenza vaccinations, including GA at the time of vaccination. The NHIS database provides comprehensive information on pregnancy episodes, including delivery procedures, diagnoses of pregnancy outcomes, prenatal tests, and pregnancy complications.

The NHIS database does not contain information on GA, which limits the precision of GA estimation when using NHIS data alone. In contrast, KIRIS provides clinically verified GA at the time of maternal influenza vaccination. The two databases were linked using resident registration numbers prior to anonymization, allowing accurate matching of vaccination records to pregnancy claims. This linkage enabled the use of KIRIS as a gold standard to validate GA algorithms derived from claims data.

#### Identification of study cohort and pregnancy-related records

In this study, pregnancy episodes rather than pregnant women were used as the unit of analysis because each pregnancy represents an independent clinical event, and the NHIS database records pregnancy outcomes, prenatal tests, and complications at the episode level. Pregnancy episodes were identified using predefined codes for pregnancy outcomes from NHIS claims data between January 1, 2018 and June 30, 2022. The codes for pregnancy outcomes are listed in [3] (Supplementary Material 1). To account for potential inaccuracies or duplications in the data, a hierarchical algorithm was applied to identify distinct pregnancy episodes (Supplementary Material 2). Pregnancy outcomes were classified into five mutually exclusive categories: live birth, stillbirth, termination, spontaneous abortion, and ectopic pregnancy. Episodes with pregnancy outcomes occurring between October 15, 2019 and March 31, 2022 were included. Episodes without influenza vaccination during pregnancy were excluded because GA information was unavailable in these cases. Accordingly, records not linked to KIRIS or with incomplete follow-up were excluded, and the cohort was restricted to episodes with at least one influenza vaccination record during the same pregnancy episode. Lastly, to reduce misclassification of GA estimation, the top and bottom 1% of the GA distribution within each pregnancy outcome category were trimmed. We empirically identified percentile ranges in which biologically implausible GA values sharply increased and applied trimming at these thresholds to remove outliers likely driven by administrative coding [12]. This approach is consistent with common preprocessing practices in large perinatal and administrative database studies, where implausible or extreme values are routinely excluded to prevent distortion by outliers. The database initially included 509,786 eligible pregnancy episodes, of which 351,055 were retained in the final analytic cohort after applying the exclusion criteria. A substantial proportion of non-live birth episodes was excluded due to the lack of GA information in the vaccination registry. Specifically, stillbirths decreased from 1,641 to 585 (64.3%), terminations from 1,028 to 139 (86.5%), spontaneous abortions from 61,138 to 3,667 (94.0%), and ectopic pregnancies from 2,183 to 60 (97.3%). Prenatal tests and pregnancy complications were identified from NHIS claims data based on the codes detailed in Table 1 and Table 2, respectively.

#### Gold standard GA

The gold standard GA at pregnancy outcome was calculated using the following formula: {(pregnancy end date−date of vaccination)+gestational weeks at vaccination}. Because KIRIS provides information only on gestational weeks, without days, at the time of influenza vaccination during pregnancy, gestational days were estimated using the midpoint of the week by adding 3 days to each GA estimate [13] (Supplementary Material 3).

#### GA estimation algorithms

Based on the NHIS database, GA was estimated using a deterministic algorithm that assigned a fixed GA to each pregnancy episode according to pregnancy outcome. The following gestational durations were assumed for each outcome: 39 weeks for live births, 28 weeks for stillbirths, and 10 weeks for terminations, spontaneous abortions, and ectopic pregnancies [10,14]. These algorithm-based GA estimates were compared with the gold standard GA from the KIRIS database to assess accuracy. The proportion of pregnancies with estimated GA at pregnancy end within 1 week, 2 weeks, 3 weeks, and 4 weeks of the gold standard GA was calculated, stratified by pregnancy outcome type.

### Statistical analysis

Pregnancy episodes were used as the unit of analysis. Based on gold standard GA information from the KIRIS database, GA was summarized using descriptive statistics, including frequencies and medians with interquartile ranges (IQRs), across pregnancy outcomes (live birth, stillbirth, termination, spontaneous abortion, and ectopic pregnancy), as well as across the timing of prenatal tests and diagnoses of major pregnancy complications. For pregnancy outcomes, GA distributions were plotted by outcome to evaluate the timing at which each outcome is coded in real-world settings and to assess whether these timings align with known clinical windows [9,10].

To assess algorithm-based GA accuracy, we calculated the proportion of episodes in which estimated GA at pregnancy end fell within ±1 week, ±2 weeks, ±3 weeks, and ±4 weeks of the gold standard GA, stratified by pregnancy outcome. To explore code-level heterogeneity in GA estimation, we also examined the distribution of algorithm-based GA across individual diagnostic and procedure codes within each pregnancy outcome category (Supplementary Materials 4-8), as well as for major prenatal tests and pregnancy complications (Supplementary Materials 4 and 5).

All analyses were performed using SAS version 9.4 (SAS Institute Inc., Cary, NC, USA) and Microsoft Excel (Microsoft Corp., Redmond, WA, USA).

### Ethics statement

The study protocol was exempted from review by the Institutional Review Board of Sungkyunkwan University, as only de-identified, routinely collected secondary data were used (2022-04-011).

## RESULTS

**Figure 1.**
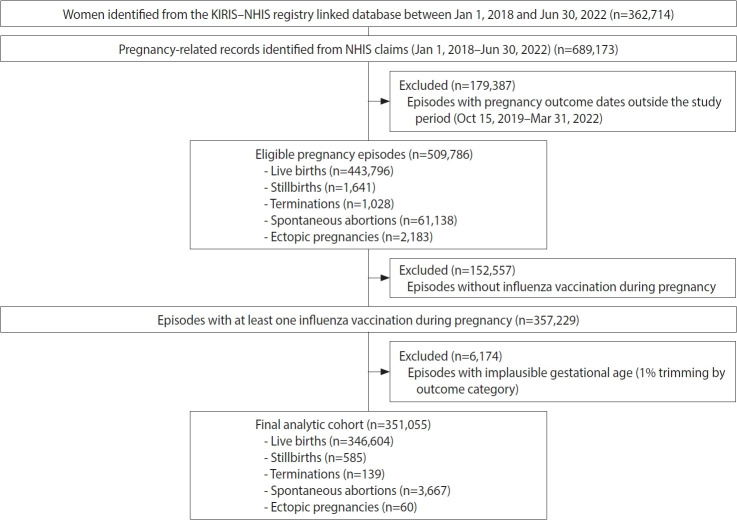
Study flow chart. KIRIS, Korea Immunization Registry Information System; NHIS, National Health Insurance Service.

**Figure 2.**
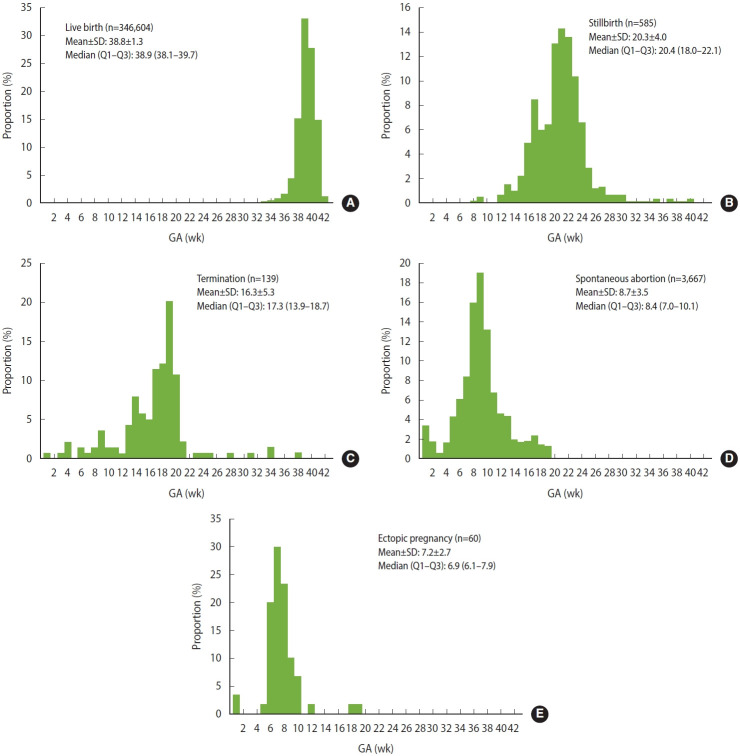
Distribution of gestational age (GA) at (A) live birth and non-live birth (B: stillbirth, C: termination, D: spontaneous abortion, and E: ectopic preg nancy) from the Korea Immunization Registry Information System–National Health Insurance Service linked database. SD, standard deviation.

Among the 351,055 pregnancy episodes identified during the study period (Figure 1), the performance of the fixed-duration GA estimation algorithm varied notably by pregnancy outcome (Table 1). For live births, algorithm-estimated GA showed high concordance with the gold standard, with 92.2% (95% confidence interval [CI], 92.1 to 92.3) of estimates falling within ±2 weeks. Accuracy further improved as wider thresholds were applied, reaching 97.3% within ±3 weeks and 98.8% within ±4 weeks. In contrast, accuracy was markedly lower for non-live birth outcomes. Stillbirths and terminations demonstrated the lowest accuracy, with only 3.3% and 7.2% of estimates within ±2 weeks, respectively, and only limited improvement at the ±4 weeks threshold (8.0 and 21.6%, respectively). For spontaneous abortions and ectopic pregnancies, approximately 45.2% and 20.0% of algorithm-based GA estimates fell within ±2 weeks, increasing to 75.2% and 73.3% within ±4 weeks, respectively. Figure 2 illustrates the distinct distribution patterns of algorithm-based GA by pregnancy outcome.

To investigate potential indicators that may serve as a foundation for improving the accuracy of algorithm-based GA estimation, we examined the gestational weeks at which prenatal tests, pregnancy complications, and preterm births were documented in the claims data, using the gold standard GA (Tables 2 and 3). Most tests were conducted within clinically expected timeframes. First-trimester general ultrasonography was commonly performed at a median of 6.8 weeks (IQR, 5.6–8.4), and first-trimester detailed ultrasonography at a median of 12.4 weeks (IQR, 11.9–12.6). Second-trimester detailed ultrasonography clustered around a median of 21.7 weeks (IQR, 20.9–23.4), and glucose tolerance testing (50 and 100 g) peaked at a median of 26.4 weeks (IQR, 25.4–27.3). Serum marker tests, such as inhibin A and alpha-fetoprotein, were recorded at a median of 16.3 weeks (IQR, 15.4–16.6). Serum β-hCG testing, which is typically performed in early pregnancy, occurred at a median of 16.1 weeks (IQR, 9.4–16.4). Pregnancy complications and preterm birth–related diagnoses also demonstrated temporal alignment with clinical expectations (Table 3). Pre-eclampsia diagnoses peaked at a median of 35.1 weeks (IQR, 31.9–37.1), gestational diabetes at 27.6 weeks (IQR, 25.4–32.6), and preterm labor and delivery at 32.3 weeks (IQR, 27.6–35.3). Hemorrhage in early pregnancy was observed at a median of 8.3 weeks (IQR, 6.6–10.7), and excessive vomiting in pregnancy at 11.0 weeks (IQR, 8.4–14.7). Late pregnancy events, including antepartum hemorrhage and placental abruption, clustered around medians of 32.3 and 37.0 weeks, respectively.

We further analyzed GA distributions for individual diagnostic and procedural codes associated with live birth and non-live birth outcomes (Supplementary Materials 6-9). For live births (Supplementary Materials 6 and 7), the dominant delivery procedure code corresponded to a median GA of 38.9 weeks (IQR, 38.3–39.7), consistent with the overall estimates shown in Table 1. Among non-live birth outcomes (Supplementary Materials 8 and 9), abortion-related codes exhibited inconsistent GA distributions, with some cases recorded beyond 30 weeks, suggesting potential misclassification. For example, the spontaneous abortion diagnosis code (O03.x) had a median GA of 6.9 weeks and a wide IQR exceeding 10 weeks, indicating substantial heterogeneity in code usage. The stillbirth diagnosis code also showed wide variability, with a median GA of 25.7 weeks (IQR, 20.4–33.6). Additional analyses of delivery characteristics (Supplementary Material 10) showed that multiple gestations were associated with earlier delivery than singleton pregnancies (median GA: 36.6 vs. 38.9 weeks). Cesarean sections were also performed at slightly earlier GAs than normal vaginal deliveries (median GA: 38.6 vs. 39.3 weeks). Although modest, these variations were consistent with known clinical patterns.

## DISCUSSION

In this study, the fixed-duration algorithm for GA estimation demonstrated high accuracy for live births but substantially lower performance for non-live birth outcomes, including stillbirth, termination, spontaneous abortion, and ectopic pregnancy. Within a ±2-week threshold, accuracy reached 92.2% for live births but was markedly lower for stillbirth (3.3%), termination (7.2%), spontaneous abortion (45.2%), and ectopic pregnancy (20.0%). Even at a ±4 weeks threshold, accuracy remained limited for stillbirth (8.0%) and termination (21.6%), indicating that fixed-duration algorithms for GA estimation exhibit pronounced outcome-specific performance limitations. In addition, findings from the distribution of pregnancy-related codes revealed substantial variability in GA estimation accuracy even when individual codes within the same outcome category were examined, highlighting the limitations of relying on fixed durations or single-code approaches.

The reduced accuracy for non-live birth outcomes may be attributed to both shorter pregnancy duration and dispersed use of diagnostic codes. Shorter gestational durations provide fewer opportunities for clinical encounters, thereby limiting the availability of informative claims-based data. Additionally, outcome-defining codes were distributed across multiple code types, reducing consistency in temporal patterns. For example, in stillbirth cases, approximately 10 different diagnostic codes were used, with no single code accounting for a majority of records; each was documented in fewer than 20% of cases [11]. This fragmentation, combined with lower data density, likely contributed to the algorithm’s underperformance in these outcome groups. Moreover, discrepancies between clinical events and their documentation in claims data—such as delayed diagnosis or delayed coding—may further reduce estimation accuracy [15,16].

Current fixed-duration GA estimation approaches often rely primarily on outcome codes, such as live birth or abortion, which limits their generalizability across heterogeneous pregnancy episodes [9]. To address this limitation, we explored additional clinical predictors that may support GA estimation. Pregnancy complications, including gestational hypertension and gestational diabetes, were recorded within clinically expected time windows, consistent with established guidelines [17,18]. Gestational diabetes was diagnosed at a median of 27.6 weeks (IQR, 25.4–32.6), aligning with the typical screening window of 24–28 weeks, whereas pre-eclampsia was recorded at a median of 35.1 weeks (IQR, 31.9–37.1), consistent with its usual onset in late pregnancy. Ultrasonography was the most frequently documented prenatal test, with second-trimester examinations clustering at 21.7 weeks (IQR, 20.9–23.4), in accordance with recommended mid-pregnancy screening intervals [19]. Some tests appeared at gestational ages that differed from recommended timing; for example, β-hCG testing frequently appeared later in our claims data, likely reflecting its use for evaluation of pregnancy-related symptoms rather than for routine early confirmation [20]. Overall, these patterns support the internal validity of algorithm-based GA estimates for anchoring time-sensitive clinical events and suggest that such markers may strengthen both deterministic and probabilistic estimation models. Previous studies have demonstrated the feasibility of integrated models for specific pregnancy groups [21,22]. More recent validation work suggests that, in the absence of delivery data, prenatal procedures and fertility-related interventions may help refine GA estimation [23]. Moreover, complementing continuous accuracy metrics with clinically meaningful GA categories, such as preterm, term, and post-term, may further aid interpretation [24]. As a potential refinement strategy, clinical predictors could be incorporated into hierarchical GA algorithms. For example, the presence of a glucose tolerance test may reasonably constrain GA to ≥24 weeks, whereas a documented first-trimester ultrasonography could restrict GA to ≤14 weeks. Combining these and other prenatal indicators may enable improved calibration of algorithm-based estimates and enhance overall accuracy in claims-based research.

This study’s primary strength lies in the use of nationwide Korean claims data linked with the immunization registry, enabling validation based on influenza vaccination records. In addition, because pharmacoepidemiologic research in Korea and many other Asian countries relies heavily on claims data, the gold-standard GA derived from the KIRIS–NHIS linkage provides an important methodological foundation for developing more reliable pregnancy-timing algorithms. This linkage has the potential to improve the accuracy and overall quality of pregnancy-related pharmacoepidemiologic studies in these settings. Nevertheless, several limitations should be considered. First, because vaccinated pregnancies may differ from unvaccinated pregnancies in health consciousness, baseline health status, and healthcare utilization, generalizability may be limited. However, prior studies have reported similar socioeconomic, clinical, and obstetric profiles between vaccinated and unvaccinated groups, suggesting that any resulting bias is likely minimal. Second, clinical context and laboratory values are not available in claims data. Third, the accuracy of diagnostic and procedure codes may vary across providers. In addition, insurance coverage policies for specific prenatal tests at designated GAs may influence when these encounters appear in claims data and should be considered when interpreting GA-related patterns. Fourth, the substantial reductions observed indicate that non-live birth outcomes are likely underrepresented in the linked dataset, which should be considered when interpreting the generalizability of findings related to these outcomes.

In conclusion, the fixed-duration algorithm demonstrated high performance for live births but limited accuracy for stillbirth, termination, spontaneous abortion, and ectopic pregnancy. Clinical features such as hypertensive disorders, gestational diabetes, ultrasonography, and β-hCG testing may improve GA estimation. Future efforts should focus on developing stratified or integrated models by pregnancy outcome to enhance GA accuracy across heterogeneous pregnancy scenarios.

## Figures and Tables

**Figure f3-epih-48-e2026007:**
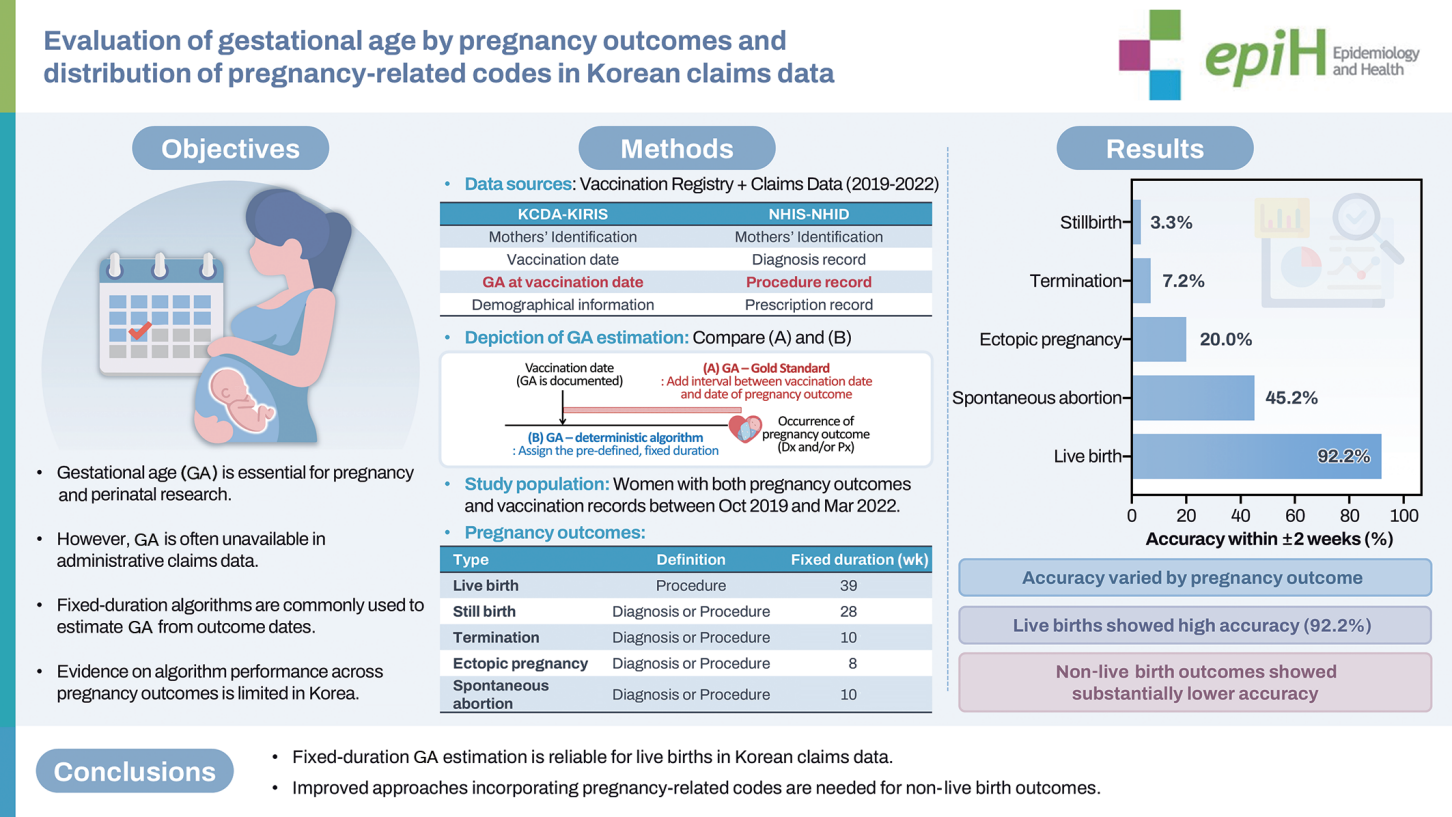


**Table 1.** Accuracy of GA estimates based on claims data compared with vaccination registry records, by pregnancy outcome

Pregnancy outcome	Median GA (Q1–Q3) Claims data	Reference GA (wk)	Absolute difference in GA between claims-based estimates and the reference standard (wk)			
			Within 1	Within 2	Within 3	Within 4
Live births (N=346,604)	38.9 (38.1–39.7)	39	64.4 (64.2, 64.5)	92.2 (92.1, 92.3)	97.3 (97.3, 97.4)	98.8 (98.8, 98.9)
Non-live births						
Stillbirth (N=585)	20.4 (18.0–22.1)	28	2.1 (1.2, 3.6)	3.3 (2.1, 5.0)	4.6 (3.2, 6.6)	8.0 (6.1, 10.5)
Termination (N=139)	17.3 (13.9–18.7)	10	3.6 (1.5, 8.1)	7.2 (4.0, 12.7)	13.7 (8.9, 20.4)	21.6 (15.6, 29.1)
Spontaneous abortion (N=3,667)	8.4 (7.9–10.1)	10	21.5 (20.2, 22.9)	45.2 (43.6, 46.8)	65.1 (63.5, 66.6)	75.2 (73.8, 76.6)
Ectopic pregnancy (N=60)	6.9 (6.1–7.9)	10	10.0 (4.7, 20.1)	20.0 (11.8, 31.8)	46.7 (34.6, 59.1)	73.3 (61.0, 82.9)

Values are presented as % (95% confidence interval). GA, gestational age; N, number of pregnancy episodes included for each outcome.

**Table 2.** Median (IQR) GA at prenatal testing, based on GA estimated from claims data

Prenatal test	GA at prenatal test			Guideline recommended midpoint (range, wk)
	N	Median	IQR (Q1–Q3)	
Ultrasonography				
Procedure				
First trimester general ultrasonography	1,050,592	6.8	2.8 (5.6–8.4)	12 (10–14)^1,2^
First trimester detailed ultrasonography	356,979	12.4	0.7 (11.9–12.6)	12 (10–14)^1,2^
Second & third trimester general ultrasonography	1,762,166	29.0	17.5 (18.4–35.9)	19 (18–20)^1,2^
Second & third trimester detailed ultrasonography	371,698	21.7	2.5 (20.9–23.4)	19 (18–20)^1,2^
Detailed fetal echocardiography	3,412	27.3	8.3 (23.6–31.9)	20 (18–22)^3^
Diagnostic code				
Antenatal screening for malformations using ultrasound and other physical methods	4,647	20.9	9.3 (12.6–21.9)	19 (18–20)^1^
Antenatal screening for fetal growth retardation using ultrasound and other physical methods	48	36.4	3.2 (34.2–37.4)	19 (18–20)^1^
Other tests				
Procedure				
Urine pregnancy test	10,492	4.9	5.4 (3.6–9.0)	No specific GA^4^
Human gene molecular genetic test	99	5.7	19.1 (0.6–19.7)	No specific GA^4^
Fetal lung maturity	68	25.8	10.5 (21.6–32.1)	No specific GA^4^
Fetal hemoglobin	37	30.6	17.7 (19.4–37.1)	No specific GA^4^
β-hCG	423,606	16.1	7.0 (9.4–16.4)	No specific GA^4^
Glucose tolerance test (50 and 100 g)	68,222	26.4	1.9 (25.4–27.3)	26 (24–28)^5^
Syphilis test	568,334	33.4	28.2 (7.4–35.6)	No specific GA^4^
Group B streptococcus	6	13.7	18.3 (5.0–23.3)	36 (35–37)^6^
Hepatitis B antigen test	531,119	30.1	28.0 (7.4–35.4)	No specific GA^4^
Inhibin A, gonadal hormone, alpha-fetoprotein	363,353	16.3	1.2 (15.4–16.6)	18 (15–20)^1^
Diagnostic code				
Pre-existing type 1 diabetes mellitus	539	25.0	18.1 (15.6–33.7)	26 (24–28)^5^
Diabetes mellitus arising in pregnancy	207,316	29.0	7.7 (26.3–34.0)	26 (24–28)^5^
Diabetes mellitus in pregnancy, unspecified	142,918	26.3	4.3 (24.9–29.1)	26 (24–28)^5^
Antenatal screening for chromosomal anomalies	3,314	16.4	1.1 (15.7–16.9)	12 (11–13) & 18 (15–20)^1^
Antenatal screening for raised alpha-fetoprotein level	98	18.8	7.0 (17.0–24.0)	18 (15–20)^1^
Other antenatal screening based on amniocentesis	64	18.5	14.6 (17.2–31.8)	18 (15–20)^3^

IQR, interquartile range; GA, gestational age; N, number of diagnostic or procedural claims for each variable; CDC, Centers for Disease Control and Prevention; ACOG, American College of Obstetricians and Gynecologists; USPSTF, United States Preventive Services Task Force.

1 CDC prenatal screening recommendations (screening for birth defects).

2 In Korea, for the first trimester, general ultrasound is reimbursed up to twice when performed at or before 13 weeks of gestation, and detailed ultrasound is reimbursed once when performed between 11 weeks and 13 weeks of gestation; For the second and third trimester, general ultrasound is reimbursed once in each of the following windows: 14–19 weeks, 20–35 weeks, and at or after 36 weeks of gestation, and detailed ultrasound is reimbursed once at or after 16 weeks of gestation.

3 Guidelines for prenatal diagnostic procedures; Recommended timing for chorionic villus sampling, amniocentesis, and fetal echocardiography follows CDC and professional society recommendations.

4 Tests without guideline-specified timing; Major clinical guidelines do not define a recommended GA window for these tests.

5 Gestational diabetes screening recommendations; Recommended timing of 24–28 weeks follows ACOG and USPSTF guidance.

6 Infection screening recommendations; Recommended timing for syphilis and hepatitis B screening at the first prenatal visit and for group B streptococcus screening at 35–37 weeks is based on CDC and ACOG guidance.

**Table 3.** Median (IQR) GA at diagnosis of pregnancy complications and preterm birth, based on GA estimated from claims data

Diagnosis	GA at diagnosis		
	N	Median	IQR (Q1–Q3)
Pregnancy complications			
Preeclampsia/eclampsia	8,296	35.1	5.2 (31.9–37.1)
Hemorrhage in early pregnancy	88,848	8.3	4.1 (6.6–10.7)
Antepartum hemorrhage	25,358	32.3	11.3 (25.0–36.3)
Excessive vomiting in pregnancy	172,251	11.0	6.3 (8.4–14.7)
Late vomiting of pregnancy	467	25.1	17.4 (13.9–31.3)
Venous complications and hemorrhoids in pregnancy	69,697	34.1	9.0 (27.7–36.7)
Gestational diabetes mellitus	349,437	27.6	7.2 (25.4–32.6)
Placental abruption	1,190	37.0	5.4 (33.3–38.7)
Oligohydramnios	12,013	37.1	4.8 (33.9–38.7)
Postpartum hemorrhage	10,534	38.9	1.6 (38.1–39.7)
Preterm birth			
Preterm labor and delivery	168,648	32.3	7.7 (27.6–35.3)
Premature rupture of membranes	62,019	37.9	6.9 (32.4–39.3)
Extreme immaturity (<28 wk)	0	N/A	N/A
Other preterm infants (≥28 to <37 wk)	3	34.3	2.3 (34.3–36.6)
Neonatal jaundice associated with preterm delivery	0	N/A	N/A

IQR, interquartile range; GA, gestational age; N, number of pregnancy episodes included for each outcome; N/A, not applicable.

## Data Availability

The data that support the findings of this study are available from the Korea Disease Control and Prevention Agency (KDCA) and the National Health Insurance Service (NHIS) but restrictions apply to the availability of these data, which were used under license for the current study, and so are not publicly available.

## Supplementary materials

Supplementary Material 1. Diagnostic and Procedural Code Definitions for Pregnancy Outcome Classification

Supplementary Material 2. Diagnostic and Procedural Code Definitions for Pregnancy Outcome Classification

Supplementary Material 3. Estimation of Gold Standard Gestational Age from the Korea Immunization Registry Information System (KIRIS) database

Supplementary Material 4. Median (IQR) Values for Gestational Age Estimation Using Procedure Codes and ICD-10 Codes for Major Prenatal Tests

Supplementary Material 5. Median (IQR) Values for Gestational Age Estimation Using ICD-10 Codes for Pregnancy -Related Diagnoses

Supplementary Material 6. Median (IQR) Values for Gestational Age Estimation Using Procedure Codes and ICD-10 Codes for Live Birth

Supplementary Material 7. Median (IQR) Values for Gestational Age Estimation Using Procedure Codes and ICD-10 Codes for Preterm Birth-Related Outcomes

Supplementary Material 8. Median (IQR) Values for Gestational Age Estimation Using Procedure Codes and ICD-10 Codes for Abortion-Related Outcomes (Including Spontaneous abortion, Termination and Ectopic pregnancy)

Supplementary Material 9. abortion, Termination and Ectopic pregnancy

Supplementary Material 10. Median (IQR) Gestational Age at Pregnancy Outcomes, Based on Gestational Age Estimated From Claims Data
