# Supplementary material for: Evaluation of gestational age by pregnancy outcomes and distribution of pregnancy-related codes in Korean claims data
Source: Epidemiol Health. 2026 Feb 4;48:e2026007. doi: 10.4178/epih.e2026007 (PMC13033438; doi:10.4178/epih.e2026007)
Supplement: Supplementary Material 1. — Diagnostic and Procedural Code Definitions for Pregnancy Outcome Classification [file epih-48-e2026007-Supplementary-1.docx]

**Supplementary Material 1.** Diagnostic and Procedural Code Definitions for Pregnancy Outcome Classification

| **Pregnancy outcome** | **ICD-10 Codes** | **Procedure Codes** |
| --- | --- | --- |
| **Live birth** |  | R3133,R3138, R3143, R3148, R4353, R4358, R4516, R4519, R4520, R5001, R5002, RA312, RA314, RA316, RA318, RA362, RA434, R3131, R3136, R3141, R3146, R4351, R4356, R4361, R4362, R4380, R4507, R4508, R4509, R4510, R4514, R4517, R4518, R4522, RA311, RA313, RA315, RA317, RA361, RA380, RA431, RA432, RA433 |
| **Stillbirth** | Z37.1x,Z37.3x, Z37.4x, Z37.6x, Z37.7x, O36.4x, O31.2x, P95.x | R4459, R4460 |
| **Termination** | O01.x, O04.x, O07.x | R4452, R4453,R4456, R4457, R4458,RY541, RY542, RY543, RY544 |
| **Spontaneous Abortion** | O02.x, O03.x, O05.x, O06.x | R4441, R4442 |
| **Ectopic pregnancy** | O00.x | R4531-R4534 |

**Abbreviation:** ICD-10, International Classification of Diseases, 10^th^ Revision. Procedure Codes, reimbursement codes for medical procedures used in Korea’s National Health Insurance Service.
