# Supplementary material for: Evaluation of gestational age by pregnancy outcomes and distribution of pregnancy-related codes in Korean claims data
Source: Epidemiol Health. 2026 Feb 4;48:e2026007. doi: 10.4178/epih.e2026007 (PMC13033438; doi:10.4178/epih.e2026007)
Supplement: Supplementary Material 2. — Diagnostic and Procedural Code Definitions for Pregnancy Outcome Classification [file epih-48-e2026007-Supplementary-2.docx]

**Supplementary Material 2.** Algorithm for identifying pregnancy episodes

1. Cohort creation

- We identified those with one or more inpatient or outpatient code for a pregnancy outcome listed in Supplementary Table 1
- We created five distinct cohort for each of the pregnancy outcomes:
  - Live birth
  - Stillbirth
  - Spontaneous abortion
  - Termination
  - Ectopic pregnancy
- In each cohort, we combined codes of the same pregnancy outcome that occurred on the same date as thy belong to the same visit. We then created 5 flags for each visit on each date with a pregnancy code as
  - Live birth (Y/N)
  - Stillbirth (Y/N)
  - Spontaneous abortion (Y/N)
  - Termination (Y/N)
  - Ectopic pregnancy (Y/N)

1. Episode creation
   - In each cohort, we ordered the visits pertaining to that particular pregnancy outcome chronologically by calendar time, and combined into one episode depending on the gap between consecutive visits. The allowable gap for each pregnancy outcome was determined after evaluating the distribution of gaps between consecutive visits. If the gap is greater than the allowable duration, the second visit is considered a separate episode
   - Allowable gaps between consecutive visits for each pregnancy outcome are:

- Live birth: 168 days
- Stillbirth: 140 days
- Spontaneous abortion: 28 days
- Termination: 28 days
- Ectopic pregnancy: 28 days
- These allowable gaps were selected based on the empirical distribution of inter-visit intervals in our dataset and are consistent with prior Korean claims-based pregnancy episode algorithms that applied clinically plausible durations to distinguish single versus separate episodes^*^.

1. Determination of pregnancy episode end date
   - For each cohort, we assigned an end date
     - Live birth: Last code for a delivery procedure code within the episode
     - Stillbirth: Last code for stillbirth within the episode
     - Spontaneous abortion: Last code for spontaneous abortion within the episode
     - Termination: Last code for termination within the episode
     - Ectopic pregnancy: Last code for ectopic pregnancy within the episode
2. Assignment of the last menstrual period (LMP)
   - In each pregnancy outcome cohort,the LMP was assigned based on a predefined gestational age (GA) duration specific to each pregnancy outcome. For each episode, we subtracted the outcome-specific GA from the episode end date to estimate the LMP. The predefined GA values used were as follows:
     - Live birth: 39 weeks
     - Stillbirth: 28 weeks
     - Spontaneous abortion: 10 weeks
     - Termination: 10 weeks
     - Ectopic pregnancy: 10 weeks
3. Combining and resolving overlapping episodes
   - Overlapping episodes with *different* pregnancy outcome types:
     - We combined all episodes belonging to the same individual and ranked them by LMP date
     - Episodes that did not overlap with others were kept as is
     - Overlapping episodes were merged into a single episode based on the following hierarchy
     - Stillbirth > Live birth > Termination > Ectopic pregnancy > Spontaneous abortion
   - Overlapping episodes with the *same* pregnancy outcome type:
     - For live births, stillbirths, and ectopic pregnancies, both episodes were retained, and the LMP of the second episode was adjusted to one day after the end date of the first episode
     - For spontaneous abortions and terminations, only the first episode was retained, and subsequent overlapping episodes were removed

*: Jung Y-S et al. *Identifying pregnancy episodes and estimating the last menstrual period using an administrative database in Korea.* Epidemiol Health. 2023.;
