# Supplementary material for: Evaluation of gestational age by pregnancy outcomes and distribution of pregnancy-related codes in Korean claims data
Source: Epidemiol Health. 2026 Feb 4;48:e2026007. doi: 10.4178/epih.e2026007 (PMC13033438; doi:10.4178/epih.e2026007)
Supplement: Supplementary Material 3. — Estimation of Gold Standard Gestational Age from the Korea Immunization Registry Information System (KIRIS) database [file epih-48-e2026007-Supplementary-3.docx]

**Supplementary Material 3.** Estimation of Gold Standard Gestational Age from the Korea Immunization Registry Information System (KIRIS) database

**
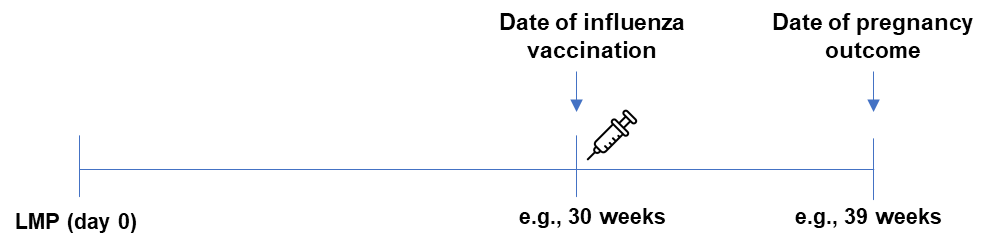
**

For example, in the case of a pregnant woman who received an influenza vaccination at 30 weeks and delivered at 39 weeks, gestational day was calculated using the formula:

Gestational day ={(pregnancy end date – date of vaccination) + Gestational weeks at vaccination} = (39 weeks – 30 weeks) + 30 weeks + 3 day = 39 weeks 3 days

KIRIS provides gestational age in completed weeks only, without information on days. To approximate the gestational day more accurately, we added 3 days, corresponding to the midpoint of each gestational week, which is a commonly used method in week-based GA data.

**Abbreviations:** LMP, last menstrual period.
