# Supplementary material for: Evaluation of gestational age by pregnancy outcomes and distribution of pregnancy-related codes in Korean claims data
Source: Epidemiol Health. 2026 Feb 4;48:e2026007. doi: 10.4178/epih.e2026007 (PMC13033438; doi:10.4178/epih.e2026007)
Supplement: Supplementary Material 4. — Median (IQR) Values for Gestational Age Estimation Using Procedure Codes and ICD-10 Codes for Major Prenatal Tests [file epih-48-e2026007-Supplementary-4.docx]

**Supplementary Material 4.** Median (IQR) Values for Gestational Age Estimation Using Procedure Codes and ICD-10 Codes for Major Prenatal Tests

| **Type of tests** | **Description** | **Timing of diagnosis/procedure** | | |
| --- | --- | --- | --- | --- |
|  |  | **N** | **Median** | **IQR (q1, q3)** |
| **Ultrasonography** | |  |  |  |
| ***Procedure code*** | |  |  |  |
| E9471^b^ | First Trimester General Ultrasonography | 11 | 7.7 | 1.4 (6.9, 8.3) |
| E9473^b^ | First Trimester General Ultrasonography | 1 | 20.4 | 0.0 (20.4, 20.4) |
| EB511^a^ | Diagnostic Ultrasonography-Obstetric Patient-First Trimester-General | 995,913 | 7.0 | 2.9 (5.7, 8.6) |
| EB512^a^ | Diagnostic Ultrasonography - Obstetric Patient - First Trimester - Pregnancy Confirmation Only | 54,780 | 5.4 | 1.1 (4.9, 6.0) |
| EB513^a^ | Diagnostic Ultrasonography - Obstetric Patient - First Trimester - Detailed | 353,970 | 12.4 | 0.7 (11.9, 12.6) |
| EB514^a^ | Diagnostic Ultrasonography - Obstetric Patient - First Trimester - Detailed (Anomaly Measurement Included) | 3,039 | 12.3 | 1.3 (11.4, 12.7) |
| E9472^b^ | Second & Third Trimester General Ultrasonography | 6 | 17.0 | 5.1 (14.3, 19.4) |
| E9474^b^ | Second & Third Trimester General Ultrasonography | 3 | 22.7 | 3.7 (20.6, 24.3) |
| EB515^a^ | Diagnostic Ultrasonography - Obstetric Patient - Second & Third Trimester - General | 1,630,496 | 28.9 | 17.3 (18.4, 35.7) |
| EB516^a^ | Diagnostic Ultrasonography - Obstetric Patient - Second & Third Trimester - High-Risk Pregnancy | 135,988 | 30.0 | 15.7 (20.7, 36.4) |
| EB517^a^ | Diagnostic Ultrasonography - Obstetric Patient - Second & Third Trimester - Detailed | 363,714 | 21.7 | 2.4 (20.9, 23.3) |
| EB518^a^ | Diagnostic Ultrasonography - Obstetric Patient - Second & Third Trimester - Detailed (Anomaly Measurement Included) | 8,133 | 23.0 | 4.4 (21.4, 25.9) |
| EB436 ^a^ | Detailed Fetal Echocardiography | 3,412 | 27.3 | 8.3 (23.6, 31.9) |
| ***ICD-10 codes*** |  |  |  |  |
| Z36.3 | Antenatal screening for malformations using ultrasound and other physical methods | 4,647 | 20.9 | 9.3 (12.6, 21.9) |
| Z36.4 | Antenatal screening for fetal growth retardation using ultrasound and other physical methods | 48 | 36.4 | 3.2 (34.2, 37.4) |
| **Other Tests** |  |  |  |  |
| D5701^c^ | Urine Pregnancy Test | 10,412 | 4.9 | 5.5 (3.5, 9.0) |
| D5702 ^c^ | Urine Pregnancy Test | 82 | 5.4 | 3.3 (4.0, 7.3) |
| D5750^c^ | Serum hCG Test | - | - | - |
| D5711^c^ | Premature Rupture of Membrane Test | 56,154 | 33.0 | 14.4 (23.6, 38.0) |
| D5712^c^ | Premature Rupture of Membrane Test | 211 | 29.7 | 7.0 (25.7, 32.7) |
| C6001^d^ | Human Gene Molecular Genetic Testing | 81 | 5.7 | 18.7 (0.6, 19.3) |
| C6003^d^ | Human Gene Molecular Genetic Testing | 3 | 21.3 | 5.6 (15.9, 21.4) |
| C6006^d^ | Human Gene Molecular Genetic Testing | 15 | 1.9 | 19.6 (0.1, 19.7) |
| CY691^d^ | Human Gene Molecular Genetic Testing | 1 | 16.4 | 0.0 (16.4, 16.4) |
| CY692^d^ | Human Gene Molecular Genetic Testing | - | - | - |
| D5721^c^ | Fetal Lung Maturity | - | - | - |
| D5722^c^ | Fetal Lung Maturity | - | - | - |
| D5723^c^ | Fetal Lung Maturity | 68 | 25.8 | 10.5 (21.6, 32.1) |
| D5724^c^ | Fetal Lung Maturity | - | - | - |
| D5725^c^ | Fetal Lung Maturity | - | - | - |
| D0561^c^ | Fetal Hemoglobin | 4 | 22.4 | 11.4 (19.3, 30.7) |
| D0562^c^ | Fetal Hemoglobin | 31 | 30.6 | 17.7 (19.4, 37.1) |
| D0563^c^ | Fetal Hemoglobin | 2 | 39.8 | 0.7 (39.4, 40.1) |
| E7325^d^ | Non-Stress Test | 406,218 | 36.6 | 3.3 (34.3, 37.6) |
| D3720^c^ | β-hCG | 412,755 | 16.1 | 10.4 (6.0, 16.4) |
| D3721^c^ | β-hCG | 10,802 | 16.4 | 1.1 (15.7, 16.9) |
| D3722^c^ | β-hCG | 56 | 4.0 | 0.7 (3.7, 4.4) |
| C7352^e^ | β-hCG | - | - | - |
| F6932^d^ | 50g, 100g Glucose Tolerance Test | 406 | 26.3 | 4.0 (24.1, 28.1) |
| E7370^a^ | 50g, 100g Glucose Tolerance Test | 67,821 | 26.4 | 1.9 (25.4, 27.3) |
| D6911^c^ | Syphilis Test | 376,437 | 33.9 | 28.3 (7.4, 35.7) |
| D6912^c^ | Syphilis Test | 6,110 | 34.3 | 27.1 (8.7, 35.9) |
| D6913^c^ | Syphilis Test | 236,244 | 32.0 | 28.0 (7.4, 35.7) |
| D6921^c^ | Syphilis Test | 867 | 14.9 | 28.6 (6.7, 35.3) |
| D6922^c^ | Syphilis Test | 81 | 19.0 | 26.0 (8.6, 34.6) |
| D6923^c^ | Syphilis Test | 1,213 | 10.3 | 25.3 (11.6, 37.4) |
| D6924^c^ | Syphilis Test | 979 | 12.0 | 27.0 (8.4, 35.4) |
| D6925^c^ | Syphilis Test | 33 | 8.3 | 22.7 (7.4, 30.1) |
| D6931^c^ | Syphilis Test | 86 | 20.9 | 25.9 (11.6, 37.4) |
| D6932^c^ | Syphilis Test | - | - | - |
| B4051^e^ | Group B Streptococcus | 6 | 13.7 | 18.3 (5.0, 23.3) |
| B4143^e^ | Group B Streptococcus | - | - | - |
| B4145^e^ | Group B Streptococcus | - | - | - |
| D7001^c^ | Hepatitis B Antigen Test | 266,513 | 13.4 | 28.3 (7.1, 35.4) |
| D7003^c^ | Hepatitis B Antigen Test | 93 | 15.9 | 27.7 (7.4, 35.1) |
| D7013^c^ | Hepatitis B Antigen Test | - | - | - |
| D7015^c^ | Hepatitis B Antigen Test | 260,248 | 33.1 | 28.3 (7.4, 35.7) |
| D7016^c^ | Hepatitis B Antigen Test | - | - | - |
| D7017^c^ | Hepatitis B Antigen Test | 199 | 28.4 | 25.9 (9.7, 35.6) |
| D7022^c^ | Hepatitis B Antigen Test | 6,528 | 24.9 | 24.1 (11.0, 35.1) |
| D7023^c^ | Hepatitis B Antigen Test | 216 | 23.4 | 24.2 (10.1, 34.4) |
| D7035^c^ | Hepatitis B Antigen Test | - | - | - |
| C4801^e^ | Hepatitis B Antigen Test | 270 | 6.6 | 2.1 (5.7, 7.9) |
| C4802^e^ | Hepatitis B Antigen Test | 358 | 6.7 | 2.4 (5.7, 8.1) |
| C4803^e^ | Hepatitis B Antigen Test | - | - | - |
| C4804^e^ | Hepatitis B Antigen Test | - | - | - |
| C4811^e^ | Hepatitis B Antigen Test | 270 | 6.6 | 2.1 (5.7, 7.9) |
| C4812^e^ | Hepatitis B Antigen Test | 357 | 6.7 | 2.4 (5.7, 8.1) |
| D5730^c^ | Inhibin A, Gonadal Hormone, Alpha-fetoprotein | 315,140 | 16.4 | 0.7 (15.9, 16.6) |
| D3710^c^ | Inhibin A, Gonadal Hormone, Alpha-fetoprotein | 349,315 | 16.3 | 1.0 (15.4, 16.4) |
| D1420^c^ | Inhibin A, Gonadal Hormone, Alpha-fetoprotein | 270 | 16.4 | 1.0 (15.9, 16.9) |
| D5740^c^ | Sflt-1 | 4,194 | 32.0 | 7.4 (27.1, 34.6) |
| ***ICD-10 codes*** | |  |  |  |
| O24.0 | Pre-existing type 1 diabetes mellitus | 539 | 25.0 | 18.1 (15.6, 33.7) |
| Z36.0 | Antenatal screening for chromosomal anomalies | 3,314 | 16.4 | 1.1 (15.7, 16.9) |
| Z36.1 | Antenatal screening for raised alpha-fetoprotein level | 98 | 18.8 | 7.0 (17.0, 24.0) |
| Z36.2 | Other antenatal screening based on amniocentesis | 64 | 18.5 | 14.6 (17.2, 31.8) |
| Z36.3 | Antenatal screening for malformations using ultrasound and other physical methods | 4,647 | 20.9 | 9.3 (12.6, 21.9) |
| Z36.4 | Antenatal screening for fetal growth retardation using ultrasound and other physical methods | 48 | 36.4 | 3.2 (34.2, 37.4) |
| Z36.5 | Antenatal screening for isoimmunization | 9 | 33.9 | 8.4 (28.1, 36.6) |
| Z36.8 | Other antenatal screening | 3,533 | 27.4 | 23.7 (12.0, 35.7) |
| Z36.9 | Antenatal screening, unspecified | 4,884 | 23.3 | 19.0 (16.0, 35.0) |
| O24.4 | Diabetes mellitus arising in pregnancy | 207,316 | 29.0 | 7.7 (26.3, 34.0) |
| O24.9 | Diabetes mellitus in pregnancy, unspecified | 142,918 | 26.3 | 4.3 (24.9, 29.1) |

**Abbreviation:** IQR, interquartile range; NA, not applicable; SD, standard deviation; N, number of diagnostic or procedural claims for each variable

a. Code used since 2016; b. Code used from 2013 to 2015; c. Code used since 2018; d. Code used since before 2010; e. Code used until 2018.

**Note:** Data were derived from the NHID–KDCA linked database and NHIS claims data for the period January 1, 2018 to June 30, 2022. The final analytic cohort consisted of 351,055 pregnancy episodes ;
