# Supplementary material for: Evaluation of gestational age by pregnancy outcomes and distribution of pregnancy-related codes in Korean claims data
Source: Epidemiol Health. 2026 Feb 4;48:e2026007. doi: 10.4178/epih.e2026007 (PMC13033438; doi:10.4178/epih.e2026007)
Supplement: Supplementary Material 5. — Median (IQR) Values for Gestational Age Estimation Using ICD-10 Codes for Pregnancy -Related Diagnoses [file epih-48-e2026007-Supplementary-5.docx]

**Supplementary Material 5.** Median (IQR) Values for Gestational Age Estimation Using ICD-10 Codes for Pregnancy -Related Diagnoses

| **Code** | **Description** | **Timing of diagnosis/procedure** | | |
| --- | --- | --- | --- | --- |
|  |  | **N** | **median** | **IQR (q1, q3)** |
| **Pregnancy Complication** | |  |  |  |
| ***ICD-10 codes*** | |  |  |  |
| O20.8, O20.9 | Hemorrhage in Early Pregnancy | 88,848 | 8.3 | 4.1 (6.6 to 10.7) |
| O20.8 | Other haemorrhage in early pregnancy | 24,095 | 8.0 | 4.0 (6.4, 10.4) |
| O11, O14, O15 | Preeclampsia/eclampsia | 8,296 | 35.1 | 5.2 (31.9 to 37.1) |
| O11 | Pre-eclampsia superimposed on chronic hypertension | 709 | 34.3 | 6.0 (30.4, 36.4) |
| O14 | Pre-eclampsia | 7,544 | 35.2 | 5.3 (32, 37.3) |
| O15 | Eclampsia | 74 | 35.7 | 5.6 (32.1, 37.7) |
| O21.0, O21.1, O21.8, O21.9, | Excessive Vomiting in Pregnancy | 172,251 | 11.0 | 6.3 (8.4 to 14.7) |
| O21.0 | Mild hyperemesis gravidarum | 99,261 | 11.0 | 6.4 (8.4, 14.9) |
| O21.1 | Hyperemesis gravidarum with metabolic disturbance | 49,149 | 11.1 | 6.1 (8.4, 14.6) |
| O21.8 | Other vomiting complicating pregnancy | 3,903 | 11.4 | 6.9 (8.4, 15.3) |
| O21.9 | Vomiting of pregnancy, unspecified | 20,824 | 10.9 | 6.7 (8, 14.7) |
| O21.2 | Late vomiting of pregnancy | 467 | 25.1 | 17.4 (13.9, 31.3) |
| O24.4 | Diabetes mellitus arising in pregnancy | 207,316 | 29.0 | 7.7 (26.3, 34) |
| O24.9 | Diabetes mellitus in pregnancy, unspecified | 142,918 | 26.3 | 4.3 (24.9, 29.1) |
| O22 | Venous complications and haemorrhoids in pregnancy | 69,697 | 34.1 | 9.0 (27.7, 36.7) |
| O41.0 | Oligohydramnios | 12,013 | 37.1 | 4.8 (33.9 to 38.7) |
| O44.1, O46 | Antepartum Hemorrhage | 25,358 | 32.3 | 11.3 (25.0 to 36.3) |
| O45 | Placental Abruption | 1,190 | 37.0 | 5.4 (33.3 to 38.7) |
| O72 | Postpartum hemorrhage | 10,534 | 38.9 | 1.6 (38.1 to 39.7) |
| **Presence of Subcodes for Gestational Weeks** | |  |  |  |
| ***ICD-10 codes*** | |  |  |  |
| Z34 | Supervision of normal pregnancy | 4938,472 | 22.1 | 21.1 (11.7, 32.9) |
| O34.3 | Maternal care for cervical incompetence | 36,380 | 27.6 | 12.9 (20.9, 33.7) |
| O60.0 | Preterm labour without delivery | 160,224 | 32.0 | 7.7 (27.4, 35.1) |

**Abbreviation:** IQR, interquartile range; NA, not applicable; SD, standard deviation; N, number of diagnostic or procedural claims for each variable;

**Note:** Data were derived from the NHID–KDCA linked database and NHIS claims data for the period January 1, 2018 to June 30, 2022. The final analytic cohort consisted of 351,055 pregnancy episodes ;
