# Supplementary material for: Evaluation of gestational age by pregnancy outcomes and distribution of pregnancy-related codes in Korean claims data
Source: Epidemiol Health. 2026 Feb 4;48:e2026007. doi: 10.4178/epih.e2026007 (PMC13033438; doi:10.4178/epih.e2026007)
Supplement: Supplementary Material 6. — Median (IQR) Values for Gestational Age Estimation Using Procedure Codes and ICD-10 Codes for Live Birth [file epih-48-e2026007-Supplementary-6.docx]

**Supplementary Material 6.** Median (IQR) Values for Gestational Age Estimation Using Procedure Codes and ICD-10 Codes for Live Birth

| **Code** | **Description** | **Timing of diagnosis/procedure** | | |
| --- | --- | --- | --- | --- |
|  |  | **N** | **median** | **IQR (q1, q3)** |
| ***Multiple Pregnancy*** | |  |  |  |
| ***Procedure codes*** | |  |  |  |
| R3133 | Induction-Primiparous-from Second Fetus in Multiple Pregnancy | 74 | 36.7 | 2.1 (35.3, 37.4) |
| R3138 | Induction-Multiparous-from Second Fetus in Multiple Pregnancy | 132 | 36.9 | 1.6 (36.0, 37.6) |
| R3143 | Forceps and Vacuum Delivery-Primiparous-from Second Fetus in Multiple Pregnancy | 96 | 36.5 | 1.6 (35.6, 37.1) |
| R3148 | Forceps and Vacuum Delivery Multiparous from Second Fetus in Multiple Pregnancy | 42 | 36.9 | 1.3 (36.3, 37.6) |
| R4353 | Normal-Primiparous-from Second Fetus in Multiple Pregnancy | 38 | 34.3 | 6.1 (30, 36.1) |
| R4358 | Normal- Multiparous- from Second Fetus in Multiple Pregnancy | 50 | 36.0 | 2.1 (34.7, 36.9) |
| R4516 | Cesarean Section Delivery- Multiple Pregnancy- Repeat | 844 | 36.6 | 1.6 (35.7, 37.3) |
| R4519 | Cesarean Section Delivery- Multiple Pregnancy- Initial-Primiparous | 5,756 | 36.6 | 1.7 (35.6, 37.3) |
| R4520 | Cesarean Section Delivery- Multiple Pregnancy- Initial- Multiparous | 983 | 36.6 | 1.7 (35.6, 37.3) |
| R5001 | Cesarean Hysterectomy- Multiple Pregnancy- Primiparous | 5 | 36.7 | 2.0 (35.3, 37.3) |
| R5002 | Cesarean Hysterectomy- Multiple Pregnancy- Multiparous | 2 | 37.5 | 1.3 (36.9, 38.1) |
| RA312 | Induced Labor (Including Augmented Labor) - Primiparous - Disabled Patient - From the Second Fetus in Multiple Pregnancy | - | - | - |
| RA314 | Induced Labor (Including Augmented Labor) - Multiparous - Disabled Patient - From the Second Fetus in Multiple Pregnancy | - | - | - |
| RA316 | Forceps or Vacuum Extraction - Primiparous - Disabled Patient - From the Second Fetus in Multiple Pregnancy | - | - | - |
| RA318 | Forceps or Vacuum Extraction - Multiparous - Disabled Patient - From the Second Fetus in Multiple Pregnancy | - | - | - |
| RA362 | Breech Delivery - Multiparous - Disabled Patient | - | - | - |
| RA434 | Normal Vaginal Delivery - Multiparous - Disabled Patient - From the Second Fetus in Multiple Pregnancy (Per Fetus) | - | - | - |
| ***Singleton Pregnancy*** | |  |  |  |
| ***Procedure codes*** | |  |  |  |
| R3131 | Induction-Primiparous-First Fetus | 40,231 | 39.6 | 1.4 (38.7, 40.1) |
| R3136 | Induction-Multiparous-First Fetus | 40,987 | 39.1 | 1.4 (38.4, 39.9) |
| R3141 | Forceps and Vacuum Delivery-Primiparous-First Fetus | 13,513 | 39.4 | 1.4 (38.7, 40.1) |
| R3146 | Forceps and Vacuum Delivery Multiparous First Fetus | 5,481 | 39.1 | 1.4 (38.4, 39.9) |
| R4351 | Normal-Primiparous-First Fetus | 24,811 | 39.3 | 1.4 (38.6, 40.0) |
| R4356 | Normal- Multiparous- First Fetus | 31,021 | 39.1 | 1.4 (38.4, 39.9) |
| R4361 | Breech Delivery- Primiparous | 84 | 36.1 | 3.2 (33.7, 36.9) |
| R4362 | Breech Delivery- Multiparous | 95 | 36.7 | 2.4 (35.4, 37.9) |
| R4380 | Vaginal Birth after Cesarean Section | 724 | 38.7 | 2.0 (37.7, 39.7) |
| R4507 | Cesarean Hysterectomy- First Fetus- Subtotal- Primiparous | 21 | 38.1 | 2.6 (36.4, 39.0) |
| R4508 | Cesarean Hysterectomy- First Fetus- Subtotal- Multiparous | 28 | 37.3 | 2.9 (35.1, 38.1) |
| R4509 | Cesarean Hysterectomy- First Fetus-Total- Primiparous | 19 | 38.1 | 3.3 (36.4, 39.7) |
| R4510 | Cesarean Hysterectomy- First Fetus-Total- Multiparous | 60 | 36.9 | 2.3 (35.4, 37.7) |
| R4514 | Cesarean Section Delivery-First Fetus-Repeat | 60,890 | 38.3 | 1.0 (37.7, 38.7) |
| R4517 | Cesarean Section Delivery-First Fetus-Initial-Primiparous | 120,827 | 38.9 | 1.4 (38.3, 39.7) |
| R4518 | Cesarean Section Delivery-First Fetus-Initial- Multiparous | 8,891 | 38.4 | 1.6 (37.6, 39.1) |
| R4522 | Trial of Labor Before Cesarean Section Delivery | 17,915 | 39.6 | 1.6 (38.7, 40.3) |
| RA311 | Induced Labor (Including Augmented Labor) - Primiparous - Disabled Patient - First Fetus | 2 | 40.0 | 2.0 (39.0, 41.0) |
| RA313 | Induced Labor (Including Augmented Labor) - Multiparous - Disabled Patient - First Fetus | 3 | 38.0 | 1.9 (37.9, 39.7) |
| RA315 | Forceps or Vacuum Extraction - Primiparous - Disabled Patient - First Fetus | 1 | 38.0 | 0.0 (38.0,38.0) |
| RA317 | Forceps or Vacuum Extraction - Multiparous - Disabled Patient - First Fetus | 1 | 37.0 | 0.0 (37.0,37.0) |
| RA361 | Breech Delivery - Primiparous - Disabled Patient | - | - | - |
| RA380 | Vaginal Birth After Cesarean (VBAC) - Disabled Patient | 1 | 30.0 | 0.0 (30.0,30.0) |
| RA431 | Normal Vaginal Delivery - Primiparous - Disabled Patient - First Fetus | 2 | 39.2 | 1.0 (38.7, 39.7) |
| RA432 | Normal Vaginal Delivery - Primiparous - Disabled Patient - From the Second Fetus in Multiple Pregnancy (Per Fetus) | - | - | - |
| RA433 | Normal Vaginal Delivery - Multiparous - Disabled Patient - First Fetus | 2 | 40.1 | 1.0 (39.6, 40.6) |

**Abbreviation:** IQR, interquartile range; NA, not applicable; SD, standard deviation; N, number of pregnancy episodes included for each outcome;

**Note:** Data were derived from the NHID–KDCA linked database and NHIS claims data for the period January 1, 2018 to June 30, 2022. The final analytic cohort consisted of 351,055 pregnancy episodes ;
