# Supplementary material for: Evaluation of gestational age by pregnancy outcomes and distribution of pregnancy-related codes in Korean claims data
Source: Epidemiol Health. 2026 Feb 4;48:e2026007. doi: 10.4178/epih.e2026007 (PMC13033438; doi:10.4178/epih.e2026007)
Supplement: Supplementary Material 7. — Median (IQR) Values for Gestational Age Estimation Using Procedure Codes and ICD-10 Codes for Preterm Birth-Related Outcomes [file epih-48-e2026007-Supplementary-7.docx]

**Supplementary Material 7.** Median (IQR) Values for Gestational Age Estimation Using Procedure Codes and ICD-10 Codes for Preterm Birth-Related Outcomes

| **Code** | **Description** | **Timing of diagnosis/procedure** | | |
| --- | --- | --- | --- | --- |
|  |  | **N** | **median** | **IQR (q1, q3)** |
| ***ICD-10 codes*** | | | | |
| **O60.x** | **Preterm labour and delivery** | 168,648 | 32.3 | 7.7 (27.6, 35.3) |
| **O60.0x** | **Preterm laubor without delivery** | 160,224 | 32.0 | 7.7 (27.4, 35.1) |
| O60.00 | <22 weeks | 16,955 | 19.1 | 3.6 (17.1, 35.1) |
| O60.01 | ≥22 weeks to <34 weeks | 86,372 | 30.4 | 5.1 (27.4, 32.6) |
| O60.02 | ≥34 weeks | 48,178 | 36.1 | 2.4 (35.0, 37.4) |
| O60.09 | Unspecified | 6,920 | 31.4 | 8.0 (26.4, 34.4) |
| **O60.1x** | **Preterm spontaneous labour with preterm delivery** | 5,696 | 34.7 | 3.7 (26.4, 34.4) |
| O60.10 | <22 weeks | 41 | 20.4 | 4.1 (17.6, 21.7) |
| O60.11 | ≥22 weeks to <34 weeks | 1,713 | 31.9 | 4.1 (29.1, 33.3) |
| O60.12 | ≥34 weeks | 3,457 | 35.7 | 1.9 (34.7, 36.6) |
| O60.19 | Unspecified | 174 | 33.9 | 5.3 (30.4, 35.7) |
| **O60.2x** | **Preterm spontaneous labour with term delivery** | 1,799 | 38.9 | 1.7 (37.9, 39.6) |
| O60.20 | <22 weeks | 11 | 37.9 | 2.1 (36.6, 38.7) |
| O60.21 | ≥22 weeks to <34 weeks | 49 | 33.4 | 6.9 (30.4, 37.3) |
| O60.22 | ≥34 weeks | 1,720 | 38.9 | 1.6 (38.0, 39.6) |
| O60.29 | Unspecified | 15 | 38.4 | 2.0 (37.0, 39.0) |
| **O60.3x** | **Preterm delivery without spontaneous labour** | 924 | 36.1 | 3.4 (34.2, 37.6) |
| O60.30 | <22 weeks | 30 | 24.8 | 14.7 (19.4, 34.1) |
| O60.31 | ≥22 weeks to <34 weeks | 143 | 32.3 | 3.4 (30.0, 33.4) |
| O60.32 | ≥34 weeks | 719 | 36.6 | 2.7 (35.6, 38.3) |
| O60.39 | Unspecified | 31 | 35.4 | 4.6 (32.7, 37.3) |
| **O42.x** | **Premature rupture of membranes** | 62,019 | 37.9 | 6.9 (32.4, 39.3) |
| **O42.0x** | **Onset of labour within 24 hours** | 5,853 | 38.4 | 2.6 (36.9, 39.4) |
| O42.00 | Early preterm (<34 weeks) | 506 | 31.7 | 5.6 (27.7, 33.3) |
| O42.01 | Late preterm (≥34 weeks to <37 weeks) | 1,162 | 36.1 | 2.0 (35.1, 37.1) |
| O42.02 | Full term (≥37 weeks) | 3,949 | 38.9 | 1.4 (38.1, 39.6) |
| O42.09 | Unspecified | 196 | 38.7 | 2.9 (36.9, 39.7) |
| **O42.1x** | **Onset of labour after 24 hours** | 396 | 35.9 | 5.5 (33.1, 38.6) |
| O42.10 | Early preterm (<34 weeks) | 169 | 33.0 | 6.6 (30.9, 37.4) |
| O42.11 | Late preterm (≥34 weeks to <37 weeks) | 107 | 35.3 | 2.1 (34.1, 36.3) |
| O42.12 | Full term (≥37 weeks) | 114 | 39 | 1.7 (38.1, 39.9) |
| O42.19 | Unspecified | 6 | 36.8 | 8.9 (29.9, 38.7) |
| **O42.2x** | **Labour delayed by therapy** | 377 | 32.7 | 7.4 (28.0, 35.4) |
| O42.20 | Early preterm (<34 weeks) | 197 | 29.7 | 7.4 (24.9, 32.3) |
| O42.21 | Late preterm (≥34 weeks to <37 weeks) | 78 | 35.2 | 2.1 (34.3, 36.4) |
| O42.22 | Full term (≥37 weeks) | 31 | 38.7 | 1.6 (38.1, 39.7) |
| O42.29 | Unspecified | 70 | 33.9 | 8.1 (28.7, 36.9) |
| **O42.9x** | **Unspecified** | 55,346 | 37.9 | 7.7 (31.6, 39.3) |
| O42.90 | Early preterm (<34 weeks) | 6,338 | 25.6 | 14.3 (17.4, 31.7) |
| O42.91 | Late preterm (≥34 weeks to <37 weeks) | 3,782 | 34.9 | 1.9 (34.9, 36.7) |
| O42.92 | Full term (≥37 weeks) | 1 | 23.4 | 0.0 (23.4, 23.4) |
| P07.2 | Extreme immaturity (<28 weeks) | 0 | - | - |
| P07.3 | Other preterm infants (≥28 weeks to <37 weeks) | 3 | 34.3 | 2.3 (34.3 to 36.6) |
| P59.0 | Neonatal jaundice associated with preterm delivery | 0 | - | - |

**Abbreviation:** IQR, interquartile range; NA, not applicable; SD, standard deviation; N, number of pregnancy episodes included for each outcome

**Note:** Data were derived from the NHID–KDCA linked database and NHIS claims data for the period January 1, 2018 to June 30, 2022. The final analytic cohort consisted of 351,055 pregnancy episodes;
