# Supplementary material for: Evaluation of gestational age by pregnancy outcomes and distribution of pregnancy-related codes in Korean claims data
Source: Epidemiol Health. 2026 Feb 4;48:e2026007. doi: 10.4178/epih.e2026007 (PMC13033438; doi:10.4178/epih.e2026007)
Supplement: Supplementary Material 8. — Median (IQR) Values for Gestational Age Estimation Using Procedure Codes and ICD-10 Codes for Abortion-Related Outcomes (Including Spontaneous abortion, Termination and Ectopic pregnancy) [file epih-48-e2026007-Supplementary-8.docx]

**Supplementary Material 8.** Median (IQR) Values for Gestational Age Estimation Using Procedure Codes and ICD-10 Codes for Abortion-Related Outcomes (Including Spontaneous

| **Code** | **Description** | **Timing of diagnosis/procedure** | | |
| --- | --- | --- | --- | --- |
|  |  | **N** | **median** | **IQR (q1, q3)** |
| ***Spontaneous Abortion*** | |  |  |  |
| ***Procedure codes*** | |  |  |  |
| R4441 | Dilation and Curettage (D&C) for Missed Abortion<12 weeks | 1,293 | 8.7 | 2.0 (7.7, 9.7) |
| R4442 | 12 weeks <Dilation and Curettage (D&C) for Missed Abortion | - | - | - |
| ***ICD-10 codes*** | |  |  |  |
| O02.x | Other abnormal products of conception | 3,938 | 8.7 | 4.0 (7.1, 11.1) |
| O03.x | Spontaneous abortion | 772 | 6.9 | 10.7 (1.6, 12.3) |
| O05.x | Other abortion | 68 | 5.6 | 12.1 (1.5, 13.6) |
| O06.x | Unspecified abortion | 158 | 7.6 | 12.7 (3.1, 15.9) |
| ***Termination*** | |  |  |  |
| ***Procedure codes*** | |  |  |  |
| R4452 | Induced abortion (≤8 weeks) | 11 | 7.0 | 3.9 (4.7, 8.6) |
| R4453 | Induced abortion (≤8 weeks) | 2 | 10.3 | 0.9 (9.9, 10.7) |
| R4456 | Induced abortion (>8 weeks to <12 weeks) | 32 | 10.6 | 3.7 (9.3, 13.0) |
| R4457 | Induced abortion (≥12 weeks to <16 weeks) | 82 | 14.6 | 2.0 (13.4, 15.4) |
| R4458 | Induced abortion (≥16 weeks to <20 weeks) | 271 | 18.0 | 2.3 (16.7, 19.0) |
| RY541 | Procedures for Pregnancy Termination | 316 | 12.4 | 9.4 (8.6, 18.0) |
| RY542 | Procedures for Pregnancy Termination | 133 | 17.0 | 8.9 (12.7, 21.6) |
| RY543 | Procedures for Pregnancy Termination | 121 | 20.1 | 5.0 (18.1, 23.1) |
| RY544 | Procedures for Pregnancy Termination | 24 | 37.6 | 3.8 (18.1, 23.1) |
| ***ICD-10 codes*** | |  |  |  |
| O01.x | Hydatidiform mole | 198 | 5.6 | 9.4 (4.1, 13.6) |
| O04.x | Medical abortion | 126 | 18.6 | 7.7 (13.7, 21.4) |
| O07.x | Failed attempted abortion | 7 | 13.4 | 15.1 (2.9, 18.0) |
| ***Ectopic pregnancy*** | |  |  |  |
| ***Procedure codes*** | |  |  |  |
| R4531 | Surgical treatment of ectopic pregnancy – Tubal or ovarian pregnancy | - | - | - |
| R4532 | Surgical treatment of ectopic pregnancy – Cornual pregnancy | - | - | - |
| R4533 | Surgical treatment of ectopic pregnancy – Cervical pregnancy | - | - | - |
| R4534 | Surgical treatment of ectopic pregnancy – Abdominal pregnancy | - | - | - |
| ***ICD-10 codes*** | |  |  | - |
| O00.x | Ectopic pregnancy | 60 | 7.2 | 1.8 (6.1, 7.9) |

abortion, Termination and Ectopic pregnancy)

**Abbreviation:** IQR, interquartile range; NA, not applicable; SD, standard deviation; N, number of pregnancy episodes included for each outcome

**Note:** Data were derived from the NHID–KDCA linked database and NHIS claims data for the period January 1, 2018 to June 30, 2022. The final analytic cohort consisted of 351,055 pregnancy episodes;
