# Supplementary material for: Evaluation of gestational age by pregnancy outcomes and distribution of pregnancy-related codes in Korean claims data
Source: Epidemiol Health. 2026 Feb 4;48:e2026007. doi: 10.4178/epih.e2026007 (PMC13033438; doi:10.4178/epih.e2026007)
Supplement: Supplementary Material 9. — abortion, Termination and Ectopic pregnancy [file epih-48-e2026007-Supplementary-9.docx]

**Supplementary Material 9.** Median (IQR) Values for Gestational Age Estimation Using Procedure Codes and ICD-10 Codes for Stillbirth-Related Outcomes

| **Code** | **Description** | **Timing of diagnosis/procedure** | | |
| --- | --- | --- | --- | --- |
|  |  | **N** | **median** | **IQR (q1, q3)** |
| ***Procedure codes*** | |  |  |  |
| R4459 | Induced Abortion (>20 weeks) | 282 | 21.4 | 2.0 (20.6, 22.6) |
| R4460 | Fetal Reduction Procedure | 4 | 15.5 | 5.2 (13.2, 18.4) |
| ***ICD-10 codes*** | | | | |
| Z37.1x | Single stillbirth | 57 | 26.9 | 6.9 (23.4, 30.3) |
| Z37.3x | Twins, one liveborn and one stillborn | 58 | 28.4 | 12.0 (21.1, 33.1) |
| Z37.4x | Twins, both stillborn | 3 | 20.9 | 2.1 (20.6, 22.7) |
| Z37.6x | Other multiple births, some liveborn | 1 | 36.0 | 0.0 (36.0, 36.0) |
| Z37.7x | Other multiple births, all stillborn | 2 | 31.8 | 18.1 (22.7, 40.9) |
| O36.4x | Maternal care for intrauterine death | 1,071 | 25.7 | 13.1 (20.4, 33.6) |
| O31.2x | Continuing pregnancy after intrauterine death of one fetus or more | 558 | 24.1 | 15.7 (16.6, 32.3) |
| P95.x | Fetal death of unspecified cause | 120 | 23.6 | 14.6 (17.6, 32.2) |

**Abbreviation:** IQR, interquartile range; NA, not applicable; SD, standard deviation; N, number of pregnancy episodes included for each outcome

**Note:** Data were derived from the NHID–KDCA linked database and NHIS claims data for the period January 1, 2018 to June 30, 2022. The final analytic cohort consisted of 351,055 pregnancy episodes;
