# Supplementary material for: Evaluation of gestational age by pregnancy outcomes and distribution of pregnancy-related codes in Korean claims data
Source: Epidemiol Health. 2026 Feb 4;48:e2026007. doi: 10.4178/epih.e2026007 (PMC13033438; doi:10.4178/epih.e2026007)
Supplement: Supplementary Material 10. — Median (IQR) Gestational Age at Pregnancy Outcomes, Based on Gestational Age Estimated From Claims Data [file epih-48-e2026007-Supplementary-10.docx]

**Supplementary Material 10.** Median (IQR) Gestational Age at Pregnancy Outcomes, Based on Gestational Age Estimated From Claims Data

| **Pregnancy Outcome** | **Median Gestational Age (Q1 to Q3)** | | |
| --- | --- | --- | --- |
|  | **N** | **Median** | **IQR (Q1 to Q3)** |
| ***Plurality*** | | | |
| Singleton Pregnancy | 338,585 | 38.9 | 1.6 (38.1 to 39.7) |
| Multiple Pregnancy | 8,019 | 36.6 | 1.7 (35.6 to 37.3) |
| ***Mode of Delivery*** | | | |
| Normal Delivery | 56,012 | 39.3 | 1.5 (38.4 to 39.9) |
| Induction of Labor | 99,180 | 39.4 | 1.5 (38.6 to 40.1) |
| Forceps or Vacuum Delivery | 19,059 | 39.4 | 1.5 (38.6 to 40.1) |
| Cesarean Delivery | 172,353 | 38.6 | 1.5 (37.9 to 39.4) |

**Abbreviation:** IQR, interquartile range; NA, not applicable; SD, standard deviation; N, number of pregnancy episodes included for each outcome

**Note:** Data were derived from the NHID–KDCA linked database and NHIS claims data for the period January 1, 2018 to June 30, 2022. The final analytic cohort consisted of 351,055 pregnancy episodes;
